# A model eye for fluorescent characterization of retinal cultures and tissues

**DOI:** 10.1038/s41598-023-37806-6

**Published:** 2023-07-06

**Authors:** G. Ferraro, Y. Gigante, M. Pitea, L. Mautone, G. Ruocco, S. Di Angelantonio, M. Leonetti

**Affiliations:** 1 Center for Life Nano- & Neuro-Science , Istituto Italiano di Tecnologia, Viale Regina Elena, 291, 00161 Rome, Italy; 2 D-Tails s.r.l. BCorp, Via di Torre Rossa, 66, 00165 Rome, Italy; 3 Department of Physiology and Pharmacology “V. Erspamer”, Sapienza University, Piazzale Aldo Moro 5, 00185 Rome, Italy; 4 Dipartimento di Fisica, Sapienza University, Piazzale Aldo Moro, 5, 00185 Rome, Italy; 5 Institute of Nanotechnology, Soft and Living Matter Laboratory, Consiglio Nazionale delle Ricerche (CNR-NANOTEC), Piazzale Aldo Moro 5, 00185 Rome, Italy

**Keywords:** Applied optics, Biotechnology

## Abstract

Many human neural or neurodegenerative diseases strongly affect the ocular and retinal environment showing peculiar alterations which can be employed as specific disease biomarkers. The noninvasive optical accessibility of the retina makes the ocular investigation a potentially competitive strategy for screening, thus the development of retinal biomarkers is rapidly growing. Nevertheless, a tool to study and image biomarkers or biological samples in a human-like eye environment is still missing. Here we report on a modular and versatile eye model designed to host biological samples, such as retinal cultures differentiated from human induced pluripotent stem cells and ex-vivo retinal tissue, but also suited to host any kind of retinal biomarkers. We characterized the imaging performance of this eye model on standard biomarkers such as Alexa Fluor 532 and Alexa Fluor 594.

## Introduction

Nowadays, several experimental evidence demonstrate the presence of retinal modifications in several diseases typical of the central nervous system (CNS). Indeed, as an integral part of the CNS, and sharing the same embryological origin with the brain and the spinal cord, the retina shares many mechanisms with the brain and often mirrors CNS disease and particularly, neurodegenerative processes^1,2^.

Ocular disorders exhibit features of neurodegenerative diseases such as Alzheimer’s (AD), Amyotrophic Lateral Sclerosis (ALS), and Parkinson’s disease (PD), showing peculiar alterations and pathological biomarkers at the ocular level^3–7^. For this reason, the eye is now considered a window to the brain^8^ providing a unique opportunity for the direct observation of the nervous tissue thanks to its transparency. Thus, retinal imaging for early diagnosis has been proposed for several pathologies including neurodegenerative diseases screening. There is, thus, a growing interest in the engineering of specific fluorescent retinal biomarkers to diagnose and monitor neurological conditions.

Phantom eyes (PEs) have been designed for decades^9^, and they are mainly employed in ophthalmic training or to calibrate, design, and evaluate the reliability of ophthalmoscopy devices^10–15^. In particular, PEs are made of synthetic (often plastic) materials and enable to emulate: (1) the anterior chamber^16^, (2) the reflectivity and thickness of the retina including the fovea^17,18^, (3) the optic nerve cup and nerve fiber layers^10,13^, and (4) the retinal detachment and dry age macular degeneration^19^. Furthermore, it should be noted that the limitation in resolution arises from the desire to test the optical performance of fluorescent labels or molecules within a human-like ocular environment, thus, following the eye's natural performance, numerical aperture of an eye-simulating device should be typically limited to 0.07^20^.

For example, Rowe and Zawadzki developed an anterior chamber eye model with the purpose of validating and comparing different instruments such as Anterior Chamber Ophthalmic Coherence Tomography (AC-OCT) instruments and corneal topographer^16^. Regarding the retina reflectivity and thickness, Baxi et al., developed a phantom eye to mimic the near infrared properties of each layer of the anatomic retina, using a fabrication process involving the deposition of nanoparticle-embedded silicon films through layer-by-layer spin coating technique. In their work, they also mimic the thickness and the surface topography of the foveal pit through the micro-etching technique^18^. Rowe and Zawadzki developed a phantom eye with the particular purpose to create a retinal layer model that closely mimic the reflectance and scattering coefficients of the anatomic retina. Both these phantom eye models have been thought to be imaged by the spectral bandwidth of Optical Coherence Tomography (OCT) instruments^17^. Kinkelder et al. developed an eye model, with the artificial retina based on thin multilayers silicon tissues used to quantify differences in nerve fiber layer thickness measurements by using different Spectral-Domain OCT system brands^10^.

Other PEs have been designed to test some instrument reliability of specific diagnostic techniques such as photoacoustic remote sensing before applying it in vivo on rat retina^21^. Agrawal et al. designed and tested a nanoparticle-embedded phantom within a model eye with the purpose to characterize the Point Spread Function (PSF) of retinal OCT devices^11^.

So far, the majority of the PEs in literature where designed and used for comparisons and validation of different kind of OCT devices, trying to better mimic the anatomic and optical properties of the eye, to optimize this category of instruments. Also, those models are made by synthetic tissues and can also be used for OCT training or to characterize the optical PSF. Therefore, a modular laboratory eye model, specific for the optical characterization of eye biomarkers in an ocular environment, is still absent both commercially and at the prototype level. Indeed, the commercially available PEs are not suitable for loading biological tissues or characterizing specific fluorescent retinal labels (FRL).

In fundus measurement, typically, just a small ratio of the total photons produced is delivered to the sensor, due to the limited numerical aperture of the eye^22^. Moreover, the specific signal is also mixed with the a-specific autofluorescence^23–25^. It is thus critical to assess the optical efficiency of FRL in an environment which simulates the human eye’s optical properties and also biological tissues. Therefore, thus far, a tool to host biological samples and give the possibility to study FRL in a human-like environment would be of great help, mostly to characterize retinal biomarkers efficiency and specificity, since such a device is still missing. Therefore, we present here, a Bio-Imaging Model Eye (BIME), which has the main purpose of filling this gap, hosting biological tissues such as retinal culture, or ex-vivo tissues, and that can be used to analyze FRL directly in a synthetic ophthalmic environment for laboratory experiments in the early stage of research.

BIME is designed to have the following advantages: (1) an optical system mimicking the human crystalline lens and cornea; (2) a water-tight environment, filled with deionized water to emulate optically both the humor vitreous and the humor aqueous, (3) a modular fundus that allows hosting biological samples such as retinal cultures derived from human induced pluripotent stem cells (iPSC) or extracted swine retinas, which are stained with proper fluorescent labels (i.e., Alexa Fluor™ 532 and Alexa Fluor™ 594); alternatively it can allow the loading of synthetic samples, like fluorescent beads, and (4) it has a curved sample holder mimicking retinal curvature. We will present here, also, how the BIME can be employed for the characterization of specific fluorescent molecules in the ocular environment.

We used BIME for the detection of fluorescence-labelled blood vessels of extracted swine retinas, and human retinal cultures, also demonstrating the possibility to simulate the eye tissue autofluorescence by adding out-of-focus colored plastic disks.

Finally, using BIME with biological samples placed into flat and curved holders, we showed that curved holders enhance the quality of the images limiting the aberrations and better imitating the properties of the eye. We also included in this model eye, the possibility to focus the sample along the Z-axis once loaded into a water-tight chamber.

## Results

We designed and realized a Bio-Imaging Model Eye (BIME) that is shown and described in this section. We performed several imaging measurements within the BIME such as: (1) iPSC derived human retinal cultures placed on flat glass coverslips, (2) the use of fluorescent disks behind the cell cultures to mimic the autofluorescence of the eye; (3) synthetic samples (fluorescents beads) loaded into flat and curved surfaces to show the difference in the quality of the image achieved, but also showing the possibility to use non-biological samples within it; (4) an example of fluorescent label characterization on the retinal tissue, and (5) representative images of extracted swine retinas placed onto flat coverslips and curved glass surfaces to better reproduce the fundamental eye properties, and thus increasing the quality of the observed details.

### Bio-imaging eye model design and imaging setup

**Figure 1:**
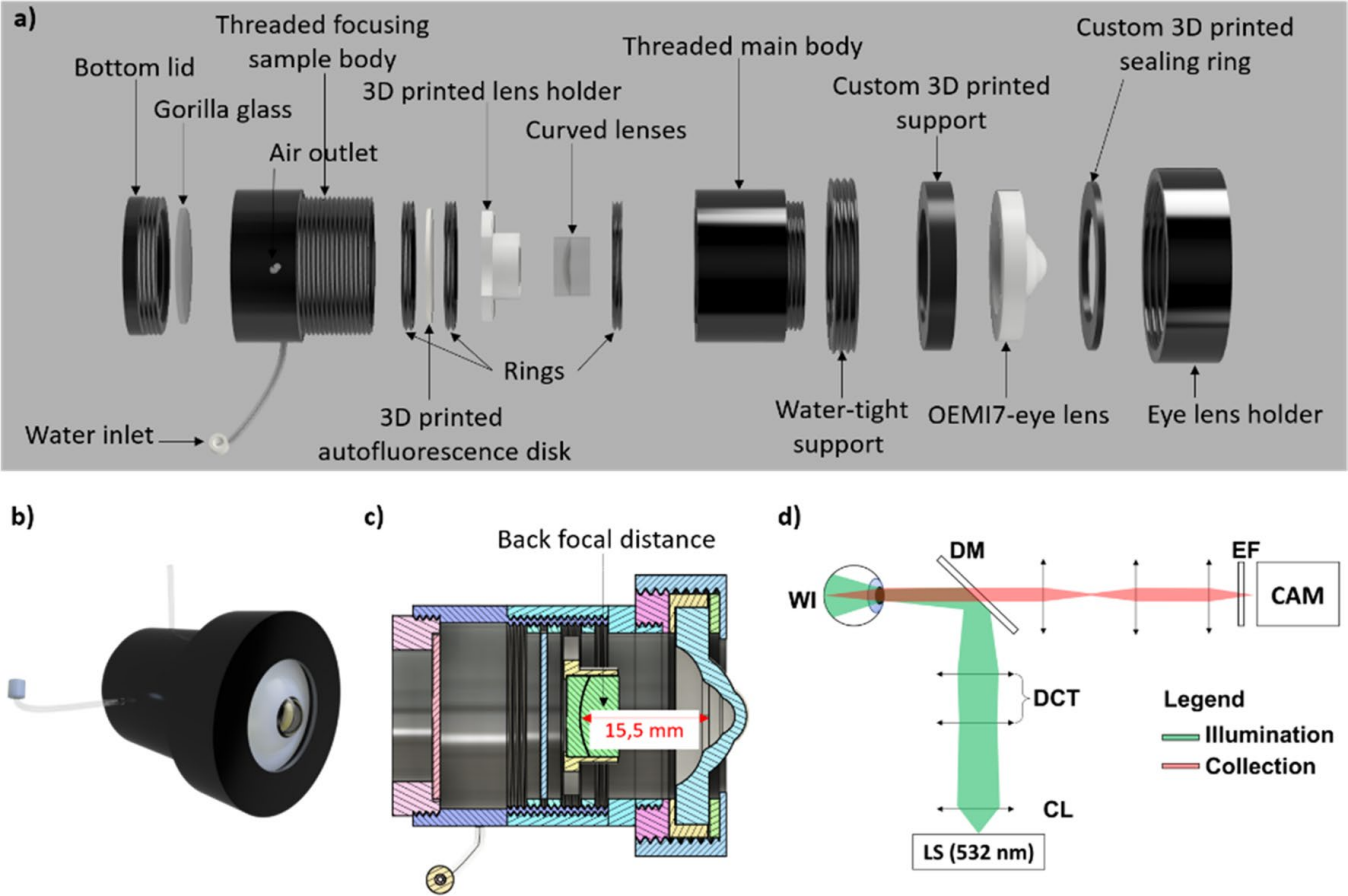
Bio-imaging Model Eye (BIME). (**a**) Shows the exploded 3D CAD model of the BIME with its components; (**b**) shows the compact view of the BIME; (**c**) reports the cross-section of it with the estimated back focal distance of the OEMI7-lens; (**d**) shows the schematic of the setup used for the imaging, where the acronyms stand for WI (Widefield Illumination), DCT (Divergence Correction Telescope), CL (Collimation Lens), LS (Light Source), DM(Dichroic Mirror), EF(Emission Filter), and CAM (CMOS Camera device).

The BIME is here reported (Fig. 1a–c), and it is composed of an ocular lens (cornea and crystalline), for a total power of 60 diopters (typical value used to indicate an emmetropic eye), and a sealed chamber. Within this chamber, Milli-Q water type I (Merck Millipore, Massachusetts, United States), with a refractive index of n = 1.333, is loaded to mimic the humor aqueous (n = 1.334) thanks to a small chamber between the cornea and the crystalline, but also the humor vitreous (n = 1.337) since it fills the space between the phantom lens and the bottom lid, directly acting as an interface between the sample and the phantom lens. Among the BIME components, we have a custom threaded sample body, that hosts the sample and allows its movements on the Z-axis different kinds of custom sample holders and disks, and a water-tight bottom with an inlet and an outlet. The inlet and the outlet were designed to easily fill the eye with pure water and to remove the excess air. Particularly the BIME uses, as the ocular lens, the lens of the commercial PE (OEMI-7, Ocular Instruments, Inc., Bellevue, WA), which has a pupil diameter of 7 mm, total power of 60 diopters, where the cornea and the crystalline are made off poly(methyl methacrylate) (PMMA with n = 1.49)^11^. However, because of the modularity of the system, adaptors for other kinds of commercial or custom synthetic lenses can be easily designed and readily 3D printed.

The external BIME body is realized by custom modular water-tight aluminum threaded parts, except for the bottom lid, which has been thought to be transparent to better inspect the presence of air bubbles trapped inside it, which could introduce some artifacts in the final image when trapped between the sample and the eye lens. Therefore, a gorilla glass (Ø 20.00 mm and 1.10 of thickness) with the coating MgF_2_ (400–700 nm) purchased from (Edmund Optics Inc., Barrington, NJ, United States) has been glued on the aluminum threaded component. Instead, the inner parts of the BIME were 3D printed with the Original Prusa i3 MK3S + (Prusa Research, Prague). Among the 3D printed parts, we have designed two different sample holders, one suitable for a round (12 mm) glass coverslips (Thorlabs, Inc., NJ, USA) with a maximum glass thickness of 0.3 mm, and the other suitable for 12.7 mm diameter glass with curved surfaces used to mimic the curvature of the retina. The curved surfaces are made by BK7 Plano-Convex and BK7 Plano-Concave lenses (EKSMA Optics Vilnius, Lithuania) which have been used as an ex-vivo swine retina holder. The Plano-Concave lens has a radius of curvature of 13 mm, a thickness of 4.00 ± 0.20 mm, and a diameter of 12.7 mm. In contrast, the Plano-Convex lens has a radius of curvature of − 13 mm, thickness of + 3.69 ± 0.20 mm, and diameter of 12.7 mm. Those lenses have a radius of curvature of about 13 mm, which value approximates the curvature value of the human retina (11–13 mm)^26,27^.

The 3D printed holders were designed to allow the air bubbles, trapped into the phantom, to be removed from the imaging area easily, thanks to specific apertures on the peripherical parts. Plastic sample holders, as well as the disks used to induce the autofluorescence, are made of PET-G (Polyethylene terephthalate glycol). In TPU (Thermoplastic Polyurethane) were 3D printed the support for centering the ocular lens (OEMI-7, Ocular Instruments, Inc., Bellevue, WA) but also the sealing ring. Both the filaments were purchased from (RS Components Srl., London, United Kingdom). The PET-G used is slightly fluorescent when shined directly with laser light at 532 nm wavelength, thus, we took advantage of this property to produce some disks, of different thicknesses, that can be used to increase the background diffusion signal and thereby mimic the autofluorescence of the retina. All the 3D-designed models are available on GitHub^28^.

As evinced from the exploded model (Fig. 1a), the sample is loaded within a threaded focusing sample holder body, and it is locked between two threaded rings. The external thread of the body allows the finely adjustment of the sample position along the Z-direction to better focus the sample. The treaded focusing body screws into the threaded main body which provided the right focusing distance between the phantom ocular lens and the sample, estimated to be ⁓ 15.5 mm as reported in the cross-section of the BIME of Fig. 1c. Figure 1b reports a 3D CAD model of the BIME.

### Biological samples and mimicked autofluorescence I

**Figure 2:**
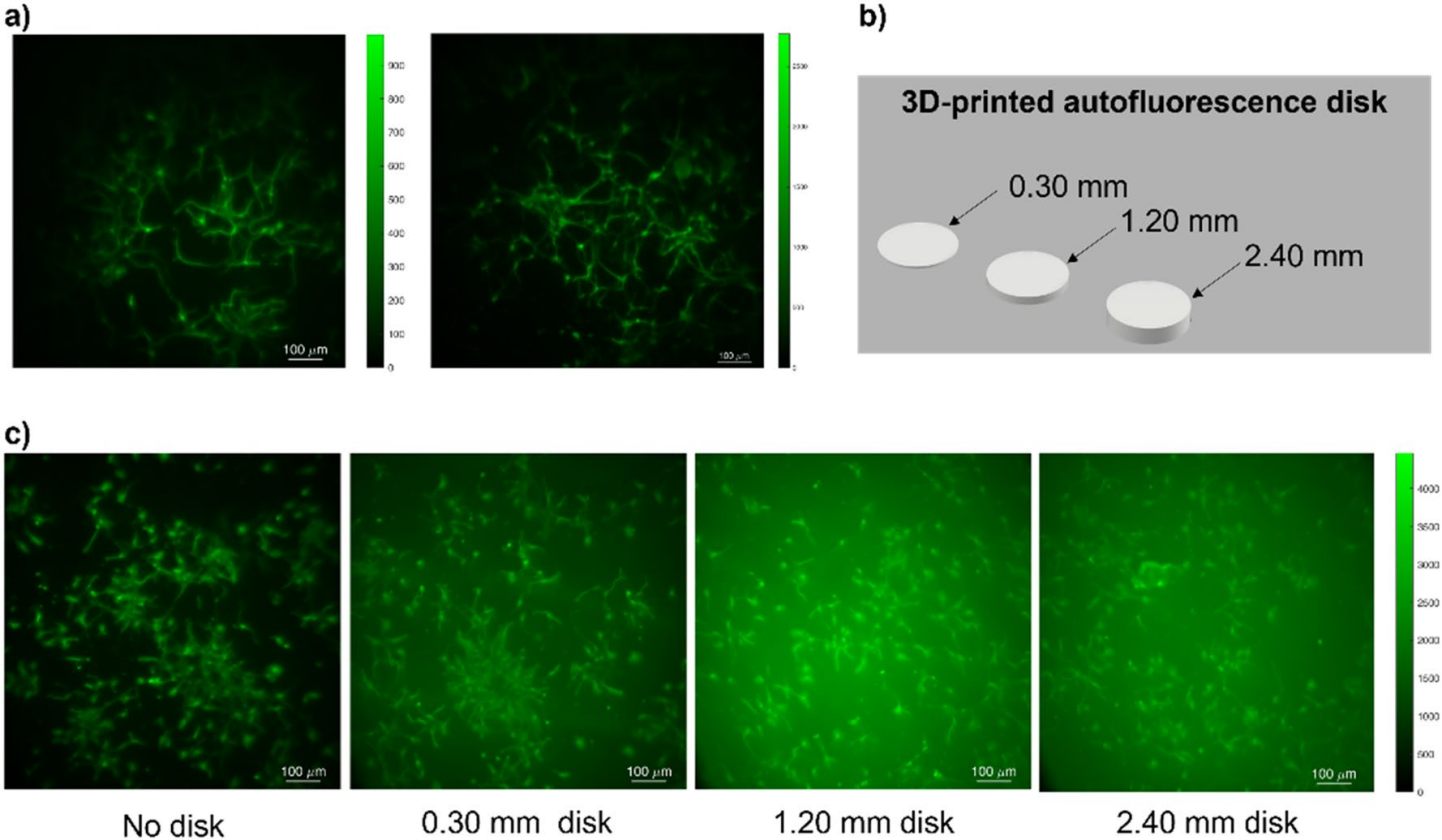
Representative images of iPSC derived retinal cultures placed in the BIME. (**a**) Shows the TUJ1 staining in differentiated iPSC-derived retinal neurons (green) at day 30 of differentiation; (**b**) shows the 3D printed disks used to mimic the autofluorescence of the retina; (**c**) shows different images of retinal neurons (green) using the autofluorescence disks placed behind the biological sample: no disc, 0.30 mm, 1.20 mm, 2.40 mm of thickness respectively from left to right, scalebar 100 µm.

We observed and reported here, representative images of biological samples plated between two flat coverslips within the BIME (Fig. 2). Figure 2a shows a representative image of the network of retinal neurons differentiated from healthy human iPSC, labeled with an antibody against TuJ1 (beta-tubulin III), a typical neuronal cytoskeletal protein (green). Note that both images are more focused in the center compared to the edge of the field. That is due to the flatness of the sample that instead needs to be curved for the optical properties of the eye.

The human eye fundus, when shined with an illumination beam, typically produces autofluorescence which can hide the faint signal from fluorescent labels. This effect is due to several factors^23^. First, as it is well known, the presence of Lipofuscin at the level of the retinal pigment epithelium^29^, induces a strong fluorescence, with the emission wavelength close to yellow-orange light^24,25^. Besides, retinoid fluorophores, in photoreceptor outer segments, are also one of the primary sources of the natural autofluorescence of the retina that can be elicited with blue light excitation (488 nm) (short-wavelength fundus autofluorescence, SW-AF)^30^.

Thus, we made a further technical implementation of the BIME, considering the overall autofluorescence. In fact, due to the modularity of the BIME, we placed behind the biological samples, specific disks mimicking the eye autofluorescence and obtaining a diffuse fluorescent signal coming from the biological sample (Fig. 2c). That has been possible since we took advantages of the properties of the white PET-G filament that fluoresces when is shined with a laser light. Figure 2b shows the disks used, particularly we try three different thicknesses (0.30, 1.20 and 2.40 respectively). Those disks were placed, specifically, behind the sample holder hosting the iPSC derived retinal culture, thus, we were able to increase the background fluorescent signal according to the thickness of the disk used (Fig. 2c). In this way, our BIME is even more representative of the human eye and can be used to train new ophthalmological devices in different operating conditions.

### Differences in imaging between flat and curved surfaces

One of the main issues of imaging samples within the BIME is that the samples need to be placed on a curved surface if the aim is to mimic the eye properties. Therefore, as observed before, placing samples between two flat surfaces results in an unfocused and aberrated image at the edge of the field. Thus, by placing samples between curved surfaces, like between two opposite curvature radius lenses (plano-concave/plano-convex lenses), it is possible to improve the result by limiting the aberrations across the field. Here we report on the difference between flat and curved surfaces used within the BIME to host the samples. Specifically, dispersions of ⁓ 2 µm fluorescent beads (Spherotech Inc, Illinois, United States) diluted in type I water, with a dilution ratio of 100:1, were used to mark the difference between the sample placed within two round glass coverslips and two curved lenses with the curvature radius (R) of R =  ± 13 mm.

**Figure 3:**
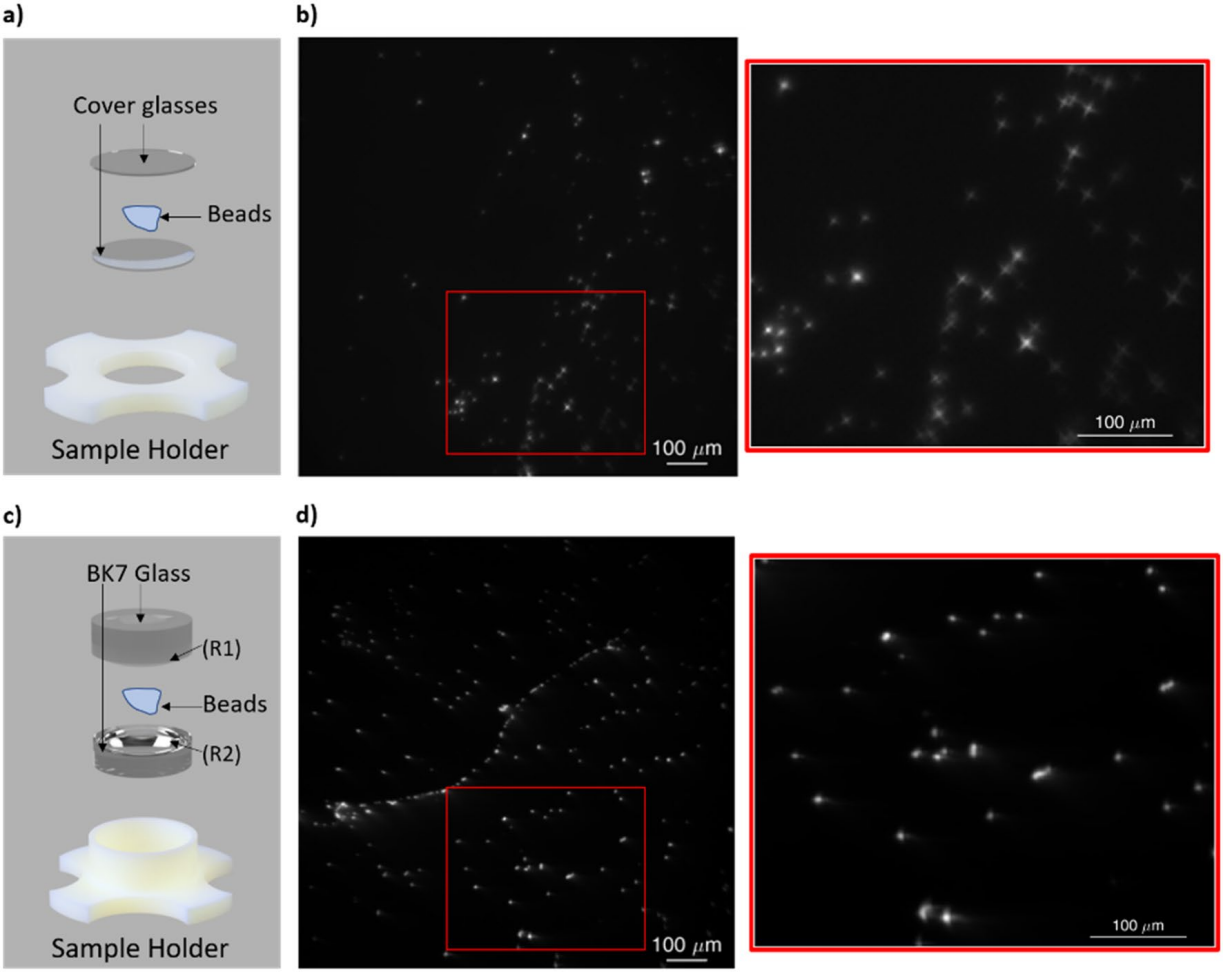
Differences between imaging on a flat and curved glass support. (**a**) Shows an illustration of the sample holder and the glass coverslips; (**b**) shows the widefield acquisition of the ⁓2 µm beads placed between two flat surfaces with the corresponding zoomed area. It is possible to notice how the aberrations rise at the edge of the field due to the flat surfaces; (**c**) reports the sample holder illustration for curved surfaces; (**d**) shows the widefield acquisition for ⁓2 µm beads placed between curved surfaces and the zoomed area, respectively. It is noticeable that the aberrations are drastically reduced. Scalebar 100 µm.

Differences between images acquired on samples placed on flat and curved surfaces are here reported (Fig. 3). Particularly, Fig. 3a and c show a schematic of the sample holder. Figure 3b shows on the left the image of the beads and on the right a zoomed area. What is striking in the figure is the difference between beads at the center and the edge of the field. The beads appear unfocused and with a star shape as we move gradually at the edge of the field; this effect is more marked by looking into the zoomed area on the right. However, using curved surfaces, as reported in Fig. 3d, aberration effects at the edge of the field are drastically reduced, as also evinced from the zoomed area on the right.

### Fluorescent retinal labels detection

In ocular environment, the number of photons detected by the camera is strongly limited by the eye's numerical aperture and aberrations. To characterize new retinal fluorescent markers, it is thus critical to quantify the total number of the photon that can be collected in an optical configuration which is as close as possible to the clinical one.

To characterize the intensity of Isolectin-labelled blood vessels of extracted swine retinas, we analyzed with BIME different samples at different incubation times with Isolectin: 40, 60, 180, 300 and 720 min respectively.

Five different extracted swine retinas were dissected and stained with Isolectin GS-IB4. At different staining time points, they were placed between flat coverslips within the BIME.

**Figure 4:**
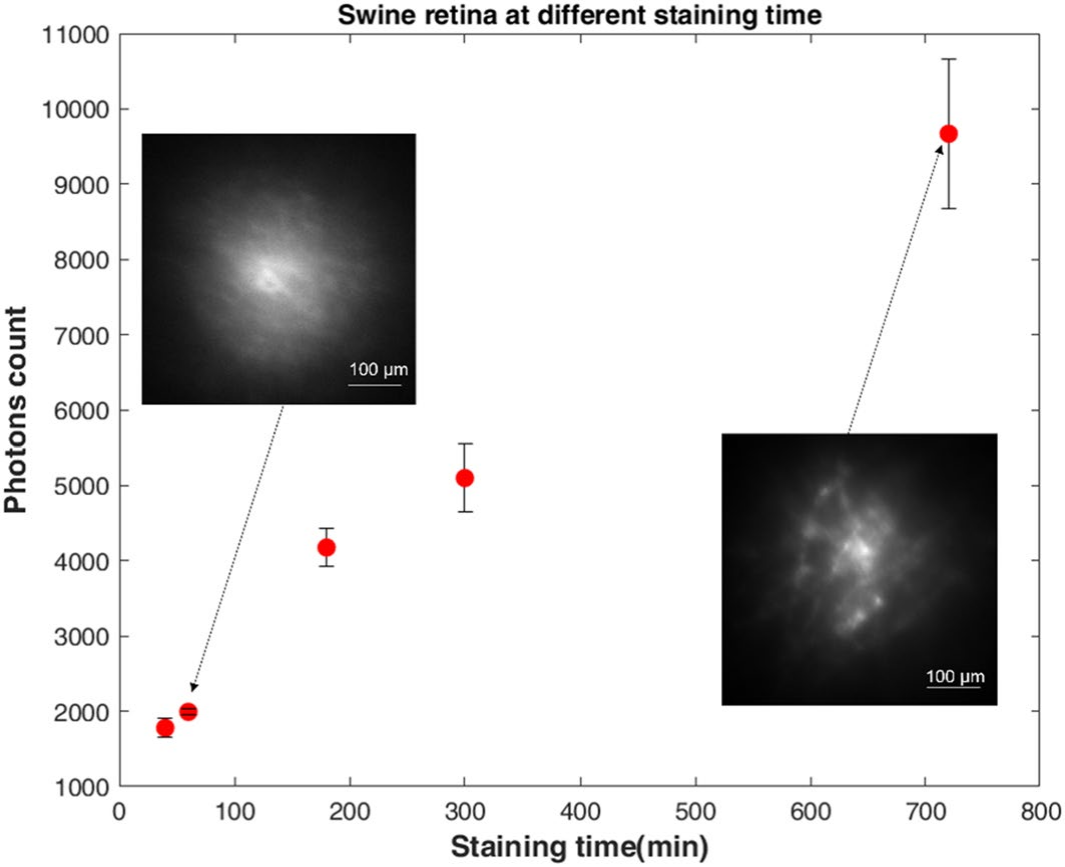
Ex-vivo retinal marker characterization as a function of the staining time. Extracted swine retinas have been stained for 40, 60, 180, 300, 720 min. Error bars were calculated with the standard error. Scalebar is 100 µm*.*

Photons count against the staining time are here reported (Fig. 4). The camera used to make the characterization was a Hamamatsu ORCA-Flash4.0 (C11440-22CU). The number of photons has been calculated following the equation:

$$P = \frac{{CF \times \left( {ADU - Off} \right)}}{Q\left( \lambda \right)/100}$$

where *P* is the Photons number, *CF* is the Conversion Factor (electron/count) and for the model of the camera used is 0.49 with the wavelength of 532 nm, *ADU* is the intensity of the pixel(s) of interest (using full 16-bit images), *Off* is the Offset count, which has the typical value around 100 and *Q* represents the Quantum Efficiency at the wavelength used.

The graph in (Fig. 4) shows a relevant increment (+ 400%) in the number of photons count over the staining time. The error bars are obtained extracting the statistical standard errors, from several samples with the same staining configuration. We conclude that overnight staining is the most suitable experimental configuration to optimal contrast in imaging Isolectin GS-IB4.

### Exploring the benefits of curved surfaces for retinal vasculature imaging

**Figure 5:**
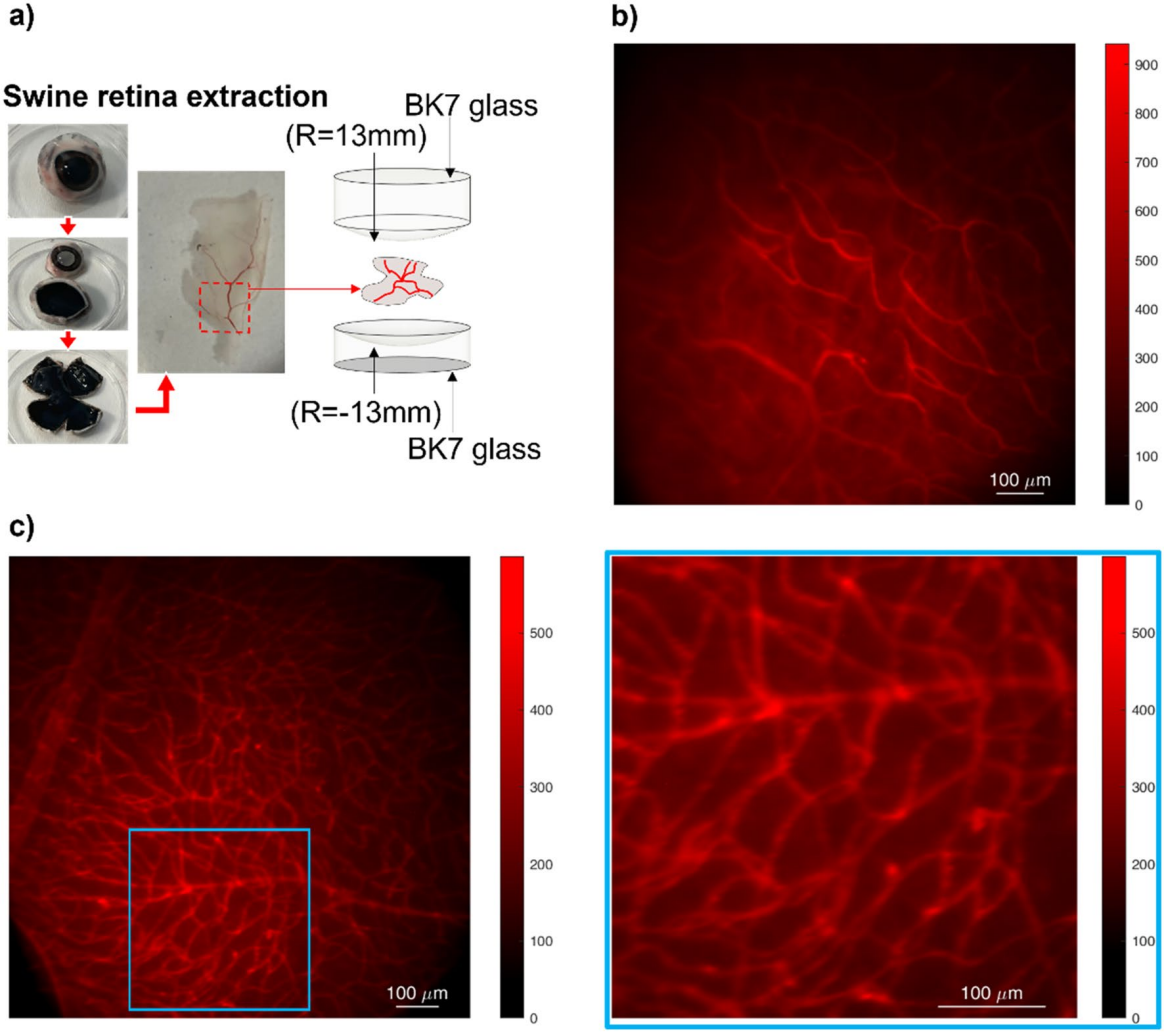
Representative images of extracted swine retina placed on flat and curved surfaces in the BI-EM. (**a**) It shows the main steps of the dissection, retina extraction, and sample loading process, specifically for the curved surfaces; (**b**) It shows an extracted swine retina placed on a flat surface within the BIEM (**c**) on the left it is reported wide field images of extracted swine retina after the overnight staining with Isolectin GS-IB4 used to label the vascular vessels (red), whereas on the right it is presented a zoomed area of the retina where is possible to notice the strong reduction of aberrations and the improvement of the focusing due to the curvature of the surface hosting the retina.

We then imaged with BIME the retinal vasculature captured in samples obtained from extracted swine retinas. Isolectin GS-IB4 staining was applied to label the blood vessels, which appeared red in the images. The samples were placed on both flat and curved surfaces (Fig. 5). The dissection procedure of the swine eye, as described in “Fluorescent retinal labels detection” section, was followed to prepare the samples. Figure 5a provides a schematic depiction of the sample preparation, while Fig. 5b,c show the acquired images. Figure 5b illustrates a retina placed in flat coverslips, whereas Fig. 5c displays the retina placed between two curved surfaces on the left and a zoomed area of it on the right. The images demonstrate that when the retina is placed between flat surfaces, not all the planes are in focus, resulting in aberration effects. However, as seen in the zoomed area of Fig. 5c, these effects are limited when the sample is placed on curved surfaces, indicating the benefits of using such surfaces for retinal vasculature imaging.

## Discussion

We developed and presented a new generation of PE. The model has been designed to study biological tissues within ocular environment, i.e., a configuration mimicking human eye’s optical properties, this peculiar characteristic makes our eye model different from the other model presented in literature or commercially available. Typically, PEs model presented in literature are designed to mimic the retina tissues synthetically in order to deliver a tool for training and provide valid OCT (or other techniques) measurements, faithfully reproducing with the anatomic structure of the eye. Also, previous PEs, have been used to test OCT devices, or to simulate specific pathologies.

The eye model we have developed serves a distinct purpose, setting it apart from other models. The BIME provides a flexible and adaptable tool primarily designed for biomarker characterization within an environment that mimics the eye. This unique model enables the inclusion of genuine biological samples or tissues, while also facilitating imaging analysis.

In our evaluation, we employed the BIME to assess its imaging capabilities on various targets, both biological and non-biological. We conducted tests using micron-sized fluorescent beads, human iPSC derived retinal neurons, and swine retina. These experiments allowed us to evaluate the imaging performance of the BIME and characterize its effectiveness across a range of subjects. The BIME demonstrates its versatility and adaptability through several distinct features. Firstly, it accommodates the usage of diverse biological samples, secondly, when employed with non-biological elements like fluorescent beads, it offers the potential to characterize the imaging performance of novel devices. Furthermore, the BIME allows for the induction of artificial autofluorescence by positioning fluorescent disks on the rear of the biological sample holder. This technique boosts the acquired diffusion signal, effectively simulating the autofluorescence observed in the retina. Additionally, the BIME serves as a valuable tool for the characterization of retinal biomarkers. Lastly, the BIME accommodates the placement of samples on both curved and flat surface. The use of curved lenses in the BIME offers a means to overcome spherical aberrations, resulting in a wider field of view for sample visualization and mimicking the behavior observed in the retina. Conversely, flat coverslips can be employed when loading biological samples onto a curved surface presents challenge.

In future iterations of the BIME, a free-standing sample holder could be incorporated, enabling the device to be employed for Optical Coherence Tomography (OCT) measurements on biological tissue. This enhancement would expand the potential applications of the BIME to encompass OCT imaging.

The versatility of the BIME extends beyond its current applications, as it has the potential to be implemented in various fields of research. It could be utilized with both healthy and diseased human or animal tissues to replicate different pathologies, thus facilitating investigations into a wide range of conditions. Importantly, the BIME holds great potential to significantly impact in-vivo imaging analyses while adhering to the principles of the 3Rs (Replacement, Reduction, and Refinement). By employing the BIME, researchers can reduce the economic and temporal burdens associated with fluorescent retinal labeling and biomarker characterization, while also considering ethical considerations. One of the key advantages of using the BIME is that it typically requires simpler ethical validation compared to full animal studies, making it a more ethically viable alternative. This adherence to ethical considerations aligns with the Replacement aspect of the 3Rs principle, aiming to reduce or replace the use of animals in scientific research whenever possible. By embracing the BIME as a substitute for traditional animal models, researchers can effectively contribute to the Reduction aspect of the 3Rs principle, reducing the number of animals needed for experimentation while still obtaining valuable insights. Furthermore, the refinement of experimental techniques through the use of the BIME allows for a more precise and controlled analysis, aligning with the Refinement component of the 3Rs principle. In summary, the adoption of the BIME in in-vivo imaging analyses not only reduces the economic and temporal burdens but also upholds the principles of the 3Rs by promoting replacement, reduction, and refinement in scientific research.

## Methods

This study involving the use of human induced pluripotent stem cells (iPSCs) adhered to the guidelines and regulations set forth by the European Union's Directive 2010/63/EU. In addition, all experimental protocols used in this study were reviewed and approved by the Ethical Committee for Transdisciplinary Research (CERT) of Sapienza University (prot.5/2022 to Silvia Di Angelantonio) in accordance with the EU's regulations on the use of iPSCs in research, ensuring that all ethical and legal considerations were met.

### Imaging setup

Images were acquired in fluorescence mode with a custom setup schematically depicted in Fig. 1d. The setup can be split into two paths: the illumination path and the collection path. The first has been designed to have the wide field illumination (WI) of the fundus of the eye, using a laser light source (LS) with the emission wavelength of 532 nm (OXXIUS, Lannion, France). The laser beam passes through a collimation lens (CL), and successive, in a divergence correction telescope (DCT) before being reflected from a dichroic mirror (DM) and shining light into the BIME. The second path is the collection path, which is made of a dichroic mirror (DM), a 4f lens system, an emission filter (EF), and a camera (CAM). By substituting both the Dichroic Mirror DM, and on the Emission Filter EF it is possible to tune the system to get a specific fluorescence signal or a generic autofluorescence. The camera used for these experiments was a Hamamatsu C11440-22CU (Hamamatsu Photonics K.K., Shizuoka, Japan).

### Preparation of retinal cultures

Healthy human induced-pluripotent stem cell lines (iPSC) were utilized to generate human retinal cultures, following a previously reported protocol with slight adjustments^31,32^. The multi-step differentiation process spans approximately 30 days, and by employing small molecules, it yields a uniform and evenly distributed 2D network of retinal neurons. Human iPSCs (iPSC0028, Sigma-Aldrich, St. Louis, Missouri, United States) were dissociated into individual cells using 1 × Accutase (Merck KGaA, Darmstadt, Germany) and seeded onto growth factor-reduced Matrigel-coated plates (1:100 dilution) at a density of 1000 cells/mm^2^ in mTeSR Plus supplemented with 10 µM Rock Inhibitor (RI; Peprotech, Cranbury, New Jersey, United States).For iPSC informed consent was obtained from all subjects and/or their legal guardian(s) by EBiSC (https://ebisc.org/). The progression of retinal neuron development can be categorized into three stages: the initial formation of a consistent and confluent sheet resembling neuroepithelium, which becomes apparent by day 6; the subsequent clustering of potential retinal progenitor macro-islands around day 20; and finally, the generation of intricate, branching retinal neurons by day 30. For further analysis, the retinal progenitor cells were seeded onto round cover glasses (Ø 0.12 mm, Thorlabs, Newton, New Jersey, United States) coated with PLO/Laminin (Sigma-Aldrich) at a density of 80,000 cells per glass. The neurogenic basal medium (N2B27w/oA), comprising a mixture of 50% DMEM/F12 [1:1] and 50% Neurobasal supplemented with 1% GlutaMAX Supplement, 0.1% Pen-Strep, 1% NEEA, 1% N2 Supplement, and 2% B27 Supplement w/oA (all from Thermo Fisher Scientific, Waltham, Massachusetts, USA), was enriched with specific small molecules at various time points: 1 µM Dorsomorphin and 2.5 µM IDE2 were freshly added each day from day 0 to day 6, while 10 mM Nicotinamide (all from Sigma-Aldrich) was provided until day 10 to enhance the differentiation of retinal cells. Additionally, 25 µM Forskolin (Sigma-Aldrich) was supplemented from day 0 to day 30 to stimulate the proliferation and expansion of retinal progenitor cells, and 10 µM DAPT (Prepotech) was introduced from day 20 to day 30 to promote the generation of retinal neurons. For subsequent analysis, three separate differentiations (corresponding to three biological replicates) were conducted.

### Dissection procedures for retina extraction

Retinal tissues were extracted from the dissection of swine eyes, which were collected in the early morning from the local butcher and transported within 4 h from the enucleation in PBS 1 × solution at 4 °C (PBS, Thermo Fisher Scientific Inc., Massachusetts, United States). The swine globes were rinsed into PBS solution for a couple of times before being dissected. The anterior segment of the globe was removed through an initial small incision under the cornea, the humor vitreous aspired with a plastic Pasteur pipette (VWR International, Pennsylvania, United States), and to ease the separation of the retina from the eyecup, four cuts were made in the sclera before to flip it. The optic nerve was cut away, and with the help of a little brush, the retina was transferred into a 24-well-dish containing phosphate-buffered saline to clean it. The retinal tissue was sized in small sections before being transferred and sealed into the glass holder. To avoid the contact of the biological tissue with water once loaded into the BIME, the glass holder has been sealed with nail polish (VWR International, Pennsylvania, United States).

### Immunostaining

Retinal neurons attached to PLO/Laminin-coated cover glasses were fixed with 4% paraformaldehyde (PFA, Sigma-Aldrich) for 15 min at room temperature, washed three times with 1 × PBS, permeabilized with PBS containing 0.2% Triton X-100 (Sigma-Aldrich) for 10 min and then blocked for 45 min at room temperature with a PBS solution containing 0.1% Tween-20 (Sigma-Aldrich) and 5% goat serum (Merck KGaA, Darmstadt, Germany). Cells were incubated overnight at 4 °C with the primary antibody TUJ1 (rabbit, 1:1000, T2200, Sigma, Merck KGaA, Darmstadt, Germany). The day after, the primary antibody solution was washed out, and the cells were incubated with secondary antibody Goat anti-rabbit Alexa Fluor™ 532 (1:750, Thermo Fisher Scientific Inc., Massachusetts, United States) for 1 h at room temperature. After primary and secondary antibody staining, washes were performed with PBS solution containing 0.1% Tween-20. The specificity of the staining was tested by performing control experiments in the absence of primary antibody incubation. To prepare cells for the analysis, the coverslips with attached and stained cells were sealed with a second cover glass using ProLong Diamond Antifade Mountant with DAPI (Invitrogen,Thermo Fisher Scientific Inc., Massachusetts, United States) was used to stain nuclei. The specificity of the staining was tested by performing control experiments in the absence of primary antibody incubation.

The dissected and extracted retinal sections were fixed with 4% PFA for 15 min at room temperature, rinsed in PBS, and incubated with Isolectin GS-IB4 (Alexa fluor 594, 1:200, Invitrogen) at 4 °C in PBS 1 × overnight. The day after, just before the imaging analysis, the retinal sections were washed three times in PBS 1 × and gently placed between two round flat cover glasses (Ø 0.12 mm, Thorlabs, Inc., New Jersey, United States) or two BK7 curved lenses (EKSMA Optics Vilnius, Lithuania) before being sealed with the nail polish (VWR International, Pennsylvania, USA) into the relative 3D printed holder. The retinal tissue was sealed between the cover glasses using DAKO (Fluorescent Mounting Medium, Sigma-Aldrich).

## Data Availability

Data presented in this paper are not publicly available at this time but may be obtained from the corresponding author upon reasonable request. The Bio-Imaging Model Eye (BIME) could be provided by the corresponding author for research use only upon reasonable request.
